# The Romanian forensic pscychiatry system related to the admission of patients- aspects of the criminal law

**DOI:** 10.1192/j.eurpsy.2024.1204

**Published:** 2024-08-27

**Authors:** C. E. Anghel, C. Băcilă, M. Tănase

**Affiliations:** ^1^Faculty of Medicine, The “Lucian Blaga” University; ^2^ Scientific Research Collective in Neurosciences - The “Dr. Gheorghe Preda” Clinical Hospital of Psychiatry; ^3^The “Dr. Gheorghe Preda” Clinical Hospital of Psychiatry, Sibiu, Romania

## Abstract

**Introduction:**

In the Romanian forensic psychiatric and legal system, the legislation allows people diagnosed with mental disorders and who have committed a crime, without discrimination, to come under the Criminal Code, thus applying the safety measure of medical hospitalization. Although it is a complex measure, which requires increased attention in its application, any omission on the part of the authorities could lead to the violation of various human rights. The role of this measure is to improve the mental state of perpetrators, who represent, both for them and for society, an important danger. Approaching from this perspective we can say that this legal framework defines and limits the circumstances in which this measure can be produced to prevent the violation of human rights..

**Objectives:**

The objective of this presentation was to carry out an analysis of the applying criteria for the safety measure of medical hospitalization, as well as the procedural aspects, in the national institutions where the perpetrators serve their sentences, called “psychiatric and security hospitals”

**Methods:**

In this way, in our research we wanted to discover the most frequent pathologies blamed to be the cause of crimes and determined the application of these measures.

**Results:**

All the results were evaluated and integrated according to the objective of this study.

**Conclusions:**

In parallel with this analysis, we wanted to identify the main aspects that make the activity difficult and also to be able to offer the possibility of creating some solutions to improve the forensic psychiatric and legal system.

**Disclosure of Interest:**

None Declared

